# Three-step *in vitro* digestion model for evaluating and predicting fecal odor emission from growing pigs with different dietary protein intakes

**DOI:** 10.5713/ab.21.0498

**Published:** 2022-04-22

**Authors:** Shih-Hua Lo, Ching-Yi Chen, Han-Tsung Wang

**Affiliations:** 1 Department of Animal Science and Technology, National Taiwan University, Taipei 10672, Taiwan

**Keywords:** Dietary Protein Manipulation, *In vitro* Fermentation, Odor Emission, Pig, Simulated Digestion

## Abstract

**Objective:**

The objective of this study was to select an effective *in vitro* digestion–fermentation model to estimate the effect of decreasing dietary crude protein (CP) on odor emission during pig production and to suggest potential prediction markers through *in vitro* and *in vivo* experiments.

**Methods:**

In the *in vitro* experiment, three diet formulations with different CP contents (170 g/kg, 150 g/kg, and 130 g/kg) but containing the same standardized ileal digestible essential amino acids (SID-EAA) were assessed. Each diet was evaluated by two different *in vitro* gastric-intestinal phase digestion methods (flask and dialysis), combined with fresh pig feces-ferment inoculation. Eighteen growing barrows (31.9±1.6 kg) were divided into three groups: control diet (180 g CP/kg, without SID-EAA adjustment), 170 g CP/kg diet, and 150 g CP/kg diet for 4 weeks.

**Results:**

The *in vitro* digestion results indicated that *in vitro* digestibility was affected by the gastric-intestinal phase digestion method and dietary CP level. According to the gas kinetic and digestibility results, the dialysis method showed greater distinguishability for dietary CP level adjustment. Nitrogen-related odor compounds (NH_3_-N, indole, *p*-cresol, and skatole) were highly correlated with urease and protease activity. The feeding study indicated that both EAA-adjusted diets resulted in a lower odor emission especially in *p*-cresol and skatole. Both protease and urease activity in feces were also closely related to odor emissions from nitrogen metabolism compounds.

**Conclusion:**

Dialysis digestion in the gastric-intestinal phase followed by fresh fecal inoculation fermentation is suitable for *in vitro* diet evaluation. The enzyme activity in the fermentation and the fecal samples might provide a simple and effective estimation tool for nitrogen-related odor emission prediction in both *in vitro* and *in vivo* experiments.

## INTRODUCTION

Odor formation is a critical problem during pig production. It is mainly produced by anaerobic bacterial fermentation of feed digestion residues, endogenous products, and dead intestinal bacteria from the gastrointestinal tract and pig manure. Many odorous compounds are the intermediate or final products of protein degradation, implying a connection between the fermentation of indigestible proteins in the diet and odor emission [1]. To support normal growth performance on a lower crude protein (CP) diet, crystalline amino acid supplementation is recommended to adjust the standardized ileal digestible (SID) essential amino acids (EAA) to meet the pig amino acid requirement in a low-CP diet. Under this feeding strategy, decreasing CP in the diet by 2% resulted in a 20% decrease in nitrogen emission from the pig in the growing and finishing phases and showed no effect on nitrogen retention [2]. It decreases the residual nitrogen for anaerobic fermentation in the hindgut, leading to lower nitrogen-related odor emissions.

The use of *in vitro* digestion models allows for comprehensive, inexpensive, efficient, and reproducible procedures for routine analysis of nutrient digestion in a short period. Static or dynamic *in vitro* digestion models have been applied to simulate gastric-intestinal (GI) phase digestion in many studies, but the complexity and the numerous steps involved in the *in vitro* models affect the results considerably [3,4]. The shake flask method is a stable and simple *in vitro* digestion method, but the method cannot simulate the absorption of digested products. However, the dialysis method can simulate the absorption process by dialysis membranes and provide similarity in the absorption process of nutrients *in vivo* [3]. Because animal fecal odorous compounds are mainly produced from microbial fermentation in the large intestine, an *in vitro* gut fermentation model with pig feces inoculation has been considered in some studies [5–7]. The kinetics profile and products of *in vitro* fermentation could provide some valuable parameters for predicting feedstuff utilization *in vivo* [6]. However, fewer studies on suitable digestion and fermentation combined with an *in vitro* model of pig diet evaluation.

The purpose of this study was to select a suitable *in vitro* digestion–fermentation model to estimate the effect of diet CP manipulation on odor emission *in vivo*. In addition, markers for predicting nitrogen-related odorous compound emissions *in vivo* were also investigated.

## MATERIALS AND METHODS

Two experiments, *in vivo* and *in vitro*, were performed to evaluate the effect of decreasing dietary CP levels on the odor emission from pigs. The *in vitro* experiment used two kinds of gastrointestinal enzyme digestion methods with the same fresh fecal inoculation process to establish the three-step *in vitro* digestion. Three CP level diets tested in the *in vitro* experiment (CP17 = 170 g/kg, CP15 = 150 g/kg, and CP13 = 130 g/kg) were adjusted EAA according to SID data from National Research Council (NRC, 2012) [8]. All diets (CP17, CP15, and CP13) evaluated in the *in vitro* experiment were 6 replicates, including each sampling time (12, 24, and 48 h) during the fermentation process.

To prevent considerably negative growth performance resulting from a large decrease in CP level, the *in vivo* feeding experiment applied CP17 and CP15 as treatment diets. The diet containing 180 g/kg CP without SID-EAA adjustment was fed as a control diet to investigate the effect of EAA adjustment on odor emission.

### *In vitro* experiment

Feed composition and the nutrient values of *in vitro* digestion are provided in Table 1. Three diet formulations with varied CP levels were ground and passed through a 20-mesh screen before weighing. The digestion process was based on previous studies [7], adopted with some modifications in digestion time length. To standardize the digestion process, the activity of digestion enzymes applied in the process was assayed as described by Minekus et al [9] before the experiment.

#### Simulated digestion fluids

The gastric fluid buffer (G-buffer) was prepared with 88.5 mM of NaCl, 6.6 mM of KCl, and 10 mM of HCl to match the *in vivo* ionic concentration of gastric fluid from growing pigs [7]. The pH of the buffer was adjusted to 2.5 with 0.2 M of HCl at 39°C. The intestinal fluid buffer (I-buffer) was prepared with 98.7 mM of NaCl, 16.4 mM of KCl, 170 mM NaH_2_PO_4_, and 30 mM Na_2_HPO_4_ to match the *in vivo* ionic concentration of small intestinal fluid from growing pigs [7], and the pH of this buffer was adjusted to 6.4 with 0.2 N of NaOH or HCl at 39°C.

#### Flask shaking digestion

To initiate gastric digestion in a conical flask, 5 g of feed (6 replicates) was added to 12.5 mL G-buffer with pepsin (0.4 g) from porcine gastric mucosa (EC 3.4.23.1, Sigma P7000, >250 units/mg; Sigma-Aldrich, St. Louis, MO, USA) at 39°C for 4 h in a shaking water bath at 150 rpm. The reaction was stopped by adding 0.625 g NaHCO_3_ and adjusting the pH to 6.8 with 0.2 N HCl or NaOH. In the intestinal phase digestion, 200 mL of prewarmed (39°C) I-buffer was added to the conical flask and mixed well. Next, 0.5 g pancreatin from porcine pancreas (8×USP, Sigma P7547; Sigma-Aldrich, USA) in 50 mL I-buffer was slowly added to the flask in the shaking water bath using a syringe. The intestinal phase digestion proceeded at 39°C for the following 16 h in a shaking water bath at 150 rpm. A check valve was set on the top of the flask to release the accumulated gas during the long reaction time. After the digestion process, the flask was moved to an ice bath to stop the reaction and transfer the content into a 500 mL centrifuge tube. The residue was collected by centrifugation at 8,000×g for 20 min (Model 7000; KOUBOTA, Tokyo, Japan). The precipitate was resolved in 5 mL of distilled water then frozen at −80°C overnight. All collected samples were dried by lyophilization and stored at 4°C as the fermentation substrate after weighing.

#### Dialysis digestion

Digestion enzymes and buffers applied in the dialysis digestion process were the same as those used in the flask shaking method. To initiate gastric digestion in a dialysis bag, 5 g of feed (6 replicates) was added to 12.5 mL HCl (0.2 N) with 0.4 g pepsin and transferred into a 15 cm rehydrated dialysis bag (Spectra/Pro 3 RC, MWCO: 1000 Da, diameter: 38 mm; Repligen, Rancho Dominguez, CA, USA). The bag was then trussed up and soaked in a 1 L beaker containing 500 mL prewarmed (39°C) G-buffer and another 1 L beaker containing 500 mL fresh, prewarmed (39°C) G-buffer with a small infusion pump attached to the beakers. Both beakers were placed in a 39°C water bath, and the buffer circulating rate was set at 120 mL/min between the two beakers. After 4 h of dialysis, NaHCO_3_ (0.025 g) was added to the dialysis bag to terminate the gastric phase digestion. Before the intestinal phase digestion, the dialysis bag was washed with warm distilled water, and then 5 mL filtered I-buffer with 0.5 g pancreatin (by Whatman No.1 filter paper) was added to the bag. The dialysis bag was soaked in the same dialysis equipment described in the gastric phase digestion, but the G-buffer was replaced with I-buffer. After 16 h of intestinal phase digestion, all contents of the dialysis bag were transferred to a 50 mL beaker and dried by lyophilization. The lyophilized samples were weighed and stored at 4°C as the fermentation substrate.

#### In vitro fermentation process of colon phase simulation

To simulate the colon fermentation process, fecal inoculum was prepared from the feces of growing pigs fed the control diet (CP = 180 g/kg). The fresh feces, collected immediately from five growing pigs when they defecated, was mixed well with the prewarmed anaerobic buffer solution (1:5, w/v) and the mixture was used as fecal inoculum. The anaerobic buffer solution was prepared according to the formula proposed by Williams et al [10]. To simulate colon fermentation, the freeze-dried residue after *in vitro* GI digestion was mixed with the fecal inoculum and anaerobic buffer (1:20:20, w/v/v) in a 100 mL serum bottle under CO_2_ flushing (final reaction volume was 80 mL). The fermentation was performed in a water bath at 39°C for 48 h (six replicates per treatment). Each bottle was connected to an ANKOM pressure sensor module, and the gas production was measured using a wireless ANKOM RF Gas Production System (ANKOM Technology Corporation, Fairport, NY, USA).

At the end of the incubation period, the cumulative gas production data were fitted to the multiphasic model described by Bauer et al [11] as follows:


$$\text{G}=\text{A}/[1+{\left(\text{C}/\text{t}\right)}^{\text{B}}]$$

where G is the total gas produced, A is the asymptotic gas production (mL), *B* is the switching characteristic of the curve, *C* is the time at which half of the asymptote has been reached (T_1/2_, h), and t is the time (h).

The maximum rate of gas production (Rmax) and the time at which it occurs (T_max_) were calculated according to the following equations [11]:


$$\begin{array}{l} {\text{R}}_{\text{max}}=(\text{A}\times {\left(\text{C}\right)}^{\text{B}}\times [{{\text{T}}_{\text{max}}}^{\left(-\text{B}-1\right)}])/{\left(1+({\text{C}}^{\text{B}})\times [{{\text{T}}_{\text{max}}}^{\left(-\text{B}\right)}]\right)}^{2}\\ {\text{T}}_{\text{max}}=\text{C}\times ({\left[(\text{B}-1)/(\text{B}+1)\right]}^{1/\text{B}}) \end{array}$$

To compare the effect of different *in vitro* digestion methods on digestive performance, fermentation was performed on 1 g of digestion residue in 50 mL vaccine bottles, according to the previously described fermentation operations in this study. The bottles were sealed with rubber stoppers and aluminum caps (18 replicates per treatment), without gas production measurements. After 12, 24, and 48 h of incubation, reactions in six vaccine bottles from each treatment group were terminated using an ice water bath, and the contents were collected for the following assay.

Fermented samples were collected and centrifuged at 400×g at 4°C for 5 min to settle the residual particles. The supernatant was used for the analysis of microbial CP, whereas the residue was used for the analysis of dry matter (DM) and CP. This supernatant was further centrifuged at 12,000×g at 4°C for 15 min. The supernatant obtained from the second centrifugation was used for the analysis of volatile fatty acids (VFA), odorous compounds, ammonia, esterase, amylase, protease, and urease. All samples for VFA and odorous compounds assay were kept in −70°C before analyzed, but samples for testing ammonia and enzyme activity were kept on ice and analyzed immediately. Samples for VFA assay were acidified with 25% meta-phosphoric acid (4:1) and filtered using a 0.22-μm filter (Millipore Co., Bedford, MA, USA). For the odorous compound assay, one milliliter of the supernatant fraction from the second centrifugation was mixed with 3 mL acetonitrile, and the supernatant was collected after another centrifugation at 13,500×g at 4°C for 10 min. All samples for VFA and odorous compounds were stored at −20°C until analysis.

### *In vivo* feeding experiment

Total eighteen growing Landrace×Yorkshire barrows (initial body weight = 31.9±1.6 kg) were randomly divided into three groups: i) Control diet (CP: 180 g/kg, without SID-EAA adjusted); ii) CP17 (SID-EAA adjusted) diet; and iii) CP15 (SID-EAA adjusted) diet. Animals in the same diet group were housed together in a sheltered pen. Animals were allowed to acclimate at the research facilities and fed the control diet for 10 days before starting the experiment. Feed composition and nutrient values for feeding trials are also shown in Table 1. Feed was provided twice daily (07:30 and 16:30), water was provided *ad libitum*, but the total feed weight was fixed at 2 kg/d/head. The total feed intake for each pen was recorded daily before the morning feeding, and the body weight of the individual animals was recorded weekly. Chromic oxide (393703; Merck, Darmstadt, Germany) was added at 0.5% of the diet as an external marker on the last four days of each week, and all fresh fecal samples were collected continuously for 4 days for digestibility estimation from each individual animal. NH_3_-N and enzyme activity in fecal samples were assayed immediately after collection, and the residual fecal samples were stored at −20°C for VFA and other odorous compound assays.

All animal care procedures used in this study were approved by the Institutional Animal Care and Use Committee of the National Taiwan University (approval no: NTU-107-EL-00056).

The apparent total tract digestibility (ATTD) of DM and nitrogen was calculated using indirect methods according to the chromium content in the samples.

#### Correlation between in vitro and in vivo parameters

Before the first day of the *in vivo* feeding experiment (all pigs were fed the control diet for 10 days), three fresh fecal samples collected from each treatment pen (total 9 samples) were mixed as the inoculum source to evaluate the correlation between *in vitro* fermentation products and the fecal products collected from the *in vivo* experiment. Each treatment diet (four replicates per treatment) was digested *in vitro* by the dialysis method as described previously, and two grams of the digestion residue was applied to the *in vitro* fermentation process of the colon phase. After 48 h of incubation, the fermentation products and enzyme activity of the fermentation liquid were collected and assayed. The VFA, NH_3_-N, odorous compounds, and enzyme activity were assayed in pig feces collected at day 14 and day 28 to compare the correlation of each parameter between *in vitro* and *in vivo* experiments.

### Chemical analysis

The DM (method 934.01), CP (method 954.01), total nitrogen (method 978.04), ether extract (method 920.39), neutral detergent fiber (method 2002.04), acid detergent fiber (method 973.18), and ash content (method 942.05) of the feed samples were analyzed according to the Association of Official Analytical Chemists [12] procedure. The chromium concentration in the fecal samples was determined using an atomic absorption spectrometer (AAnalyst 200; PerkinElmer Instruments, Norwalk, MA, USA). Ammonia-N was determined using a colorimetric method according to the phenol-hypochlorite reaction [2]. The pH value during the *in vitro* fermentation period was measured using a pH meter (LAQUA 1000; HORIBA, Kyoto, Japan) at 12, 24, and 48 h.

For VFA and other odorous compounds assays, one gram of fresh fecal sample was mixed with 9 mL 0.1 N H_2_SO_4_ and collected using the centrifugal supernatant (12,000×g; 15 min; 4°C) for high performance (pressure) liquid chromatography (HPLC) assay. Another one gram of fresh fecal sample was mixed with 9 mL cold phosphate buffered saline by vortex collected with the centrifugal supernatant (12,000×g; 15 min; 4°C) for NH3-N and enzyme activity assay.

Volatile fatty acids (acetic, propionic, and butyric acids) and odor compounds (p-cresol, indole, and 3-methylindole) were determined in an aliquot from the original acidified sample. For VFA analysis, the thawed sample (20 μL) was analyzed using HPLC with a UV detector (JASCO, Easton, MD, USA) at a wavelength of 210 nm. The VFAs were separated using a Rezex ROA-Organic Acid H^+^ (8%) column (300 mm×7.8 mm; Phenomenex, Torrance, CA, USA) with 0.01 N H_2_SO_4_ as the mobile phase at a flow rate of 0.5 mL/min and a column temperature of 40°C.

For the odorous compound assay, the thawed sample (10 μL) was analyzed using HPLC with a fluorescence detector (JASCO, USA). Compounds were separated using a LiChrospher 100 RP-18 (5 μm) LiChroCART 250-4 column (Merck, Burlington, MA, USA). The mobile phase was a mixture of 200 mM ammonium formate and acetonitrile (52:48), the flow rate was 0.5 mL/min, the column temperature was set at 30°C, and the excitation and emission wavelengths of the detector were 270 and 340 nm, respectively.

Microbial protease activity was analyzed by measuring the released amount of azo-group after incubating the sample with 0.8% azocasein (Sigma 11610; Sigma-Aldrich, USA) at 38°C for 30 min. The reaction was blocked by 10% trichloroacetic acid addition on ice. After centrifugation, the absorbance at 450 nm was measured.

Microbial urease and amylase were analyzed using the urease activity assay kit (K378; Biovision, Milpitas, CA, USA) and amylase assay kit (K-CERA, Megazyme, MI, USA) according to the manufacturer’s instructions. Microbial esterase assays were conducted using *p*-naphthyl acetate substrate, as described by Schmidt and Bornscheuer [13], but the buffer system was replaced with sodium phosphate buffer (50 mM, pH 6.8) to simulate the pig fecal condition.

### Statistical analysis

Statistical analyses were performed using SAS software (version 14.1; SAS, Cary, NC, USA). Data of *in vitro* fermentation results from two different digestion methods under three different sampling time (each CP level diet with six fermentation bottles) were compared using the general linear model (GLM) procedure in SAS for a 2×3 factorial treatment arrangement. The analysis of variance included the main effects of the CP level, digestion method, and CP level × digestion method interaction. The statistical model used was:


$${\text{Y}}_{\text{ijk}}=\mu +\alpha_{\text{i}}+\beta_{\text{j}}+{\left(\alpha \beta \right)}_{\text{ij}}+e_{\text{ijk}}$$

where μ is the overall mean and *e*_ijk_ is the random error term. α and β are the effects of digestion method and CP level, respectively. The effect of digestion method and interaction between digestion method and CP level was further divided into linear and quadratic effects using orthogonal polynomial contrasts. The mean values and pooled standard errors of the means were reported. The digestion and fermentation results from *in vitro* and *in vivo* experiments were analyzed using the GLM procedure of SAS. The linear and quadratic effect of CP level in the *in vivo* experiment (three diets, six pigs for one treatment) were determined by orthogonal polynomial contrasts. For metabolites, Pearson’s correlation was calculated and the analysis of the classification of means by the difference of least squares means method were performed using the CORR procedures of the SAS. The REG procedure of SAS was used to develop regression equations for *in vitro* and *in vivo* parameters. The probability level (p<0.05) was identified as statistically significant for all variables.

## RESULT

### Comparison of *in vitro* digestion methods in digestibility and fermentation parameters

The effects of the *in vitro* digestion method on gas production kinetics and fermentation products under different CP levels are shown in Table 2. According to the gas production parameters, the asymptotic gas production (A value) was not affected by the digestion method and CP level but only quadratic interaction between CP level and digestion method (p<0.001) existed. However, the characteristics of the curve (B value) and the half-time of maximum gas production (C value) were affected by the digestion method (p<0.001). The R_max_ values during the fermentation period were affected by both CP levels (p<0.001) and digestion method (p<0.001), but the T_max_ values were not affected by digestion method. The dialysis digestion method showed a greater B value and R_max_ (p<0.001). The difference in half-time and T_max_ between CP levels after the dialysis digestion method also indicated that this method could distinguish the effect of CP levels during *in vitro* gastric intestinal digestion.

The *in vitro* DM and CP disappearance were affected by the digestion method and dietary CP level (p<0.001), but higher CP levels resulted in greater *in vitro* GI phase and total DM disappearance. In all dialysis digestion treatments, the *in vitro* total CP disappearance was over 90%, but it was lower in flask digestion treatments. Notably, the CP15 diet showed lower *in vitro* GI phase and total CP disappearance in both digestion methods and quadratic effect of CP level and quadratic interaction between CP level and digestion method were shown in total CP digestibility (p<0.001), but the NH_3_-N concentration at 48 h was not associated with the *in vitro* CP digestibility (Table 3).

Both pH and NH_3_-N concentrations were higher in flask digestion treatments compared with dialysis digestion group after 12, 24, and 48 h of fermentation. Data from different sampling time also showed the effect of method, quadratic effect of CP level and quadratic interaction between CP level and digestion method on acetic acid and propionic acid (p< 0.001). However, the VFA fermentation results were very similar for the three CP levels after flask digestion.

After 48 h of fermentation, the concentrations of total VFA, acetate, and butyrate were higher in the dialysis digestion group than in the flask digestion group and increased with increasing CP level. However, similar total and individual VFA concentrations were shown among the three flask digestion treatments (Table 3). The individual VFA ratios also exhibited different trends between the two digestion methods. The calculated value of acetate to propionate ratio (Ac/Pr) ranged from 1.75 (in CP17 diet) to 3.10 (in CP13 diet) among dialysis digestion treatments, but it was closed to 1.0 in all flask digestion treatments. A higher propionate concentration was observed after flask digestion than after dialysis digestion, but the CP level had no effect on propionate concentration after flask digestion. Flask digestion caused a higher NH_3_-N concentration at the end of fermentation (p<0.001), and the decreased CP level resulted in lower NH_3_-N.

The flask digestion treatments showed higher esterase and urease activities than the dialysis digestion treatment (p<0.001), but the amylase activity showed no differences among CP levels after 24 h and 48 h of fermentation. The protease and urease activities after 48 h of fermentation were affected by CP level (p<0.001) and digestion method (p<0.001), and the quadratic effect of CP level (p = 0.021) were shown in urease activities.

**Figure 1:**
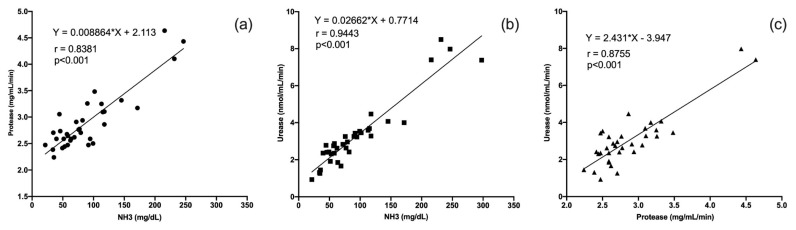
The linear relationship between (a) NH_3_-N concentration and protease activity; (b) NH_3_-N concentration and urease activity; (c) protease activity and urease activity. Data collected from each sampling time during 48 h *in vitro* fermentation (substrates from dialysis digestion method).

According to the NH_3_-N concentration measured after different fermentation times of all treatments, the NH_3_-N concentration was highly related to protease and urease activity (R^2^ = 0.84 and 0.94, respectively; Figure 1a and 1b). A high coefficient of determination was also shown in the regression analysis between protease activity and urease activity (R^2^ = 0.87; Figure 1c).

### Correlation of *in vitro* fermentation products

The correlation coefficients between *in vitro* fermentation products are shown in the upper half of Table 4. The correlation coefficients between protease and odor compounds were significant for indole (r = 0.82, p = 0.002) and NH_3_-N (r = 0.81, p<0.001). There also existed a strong relationship between urease and protease (r = 0.91, p<0.001). The results showed that the relationship between urease and odor compounds was strong for *p*-cresol (r = 0.88, p = 0.015) and NH_3_-N (r = 0.92, p<0.001). However, the correlation coefficients between enzyme activity (protease and urease) and skatole were less than 0.70. The assay results showed a significant correlation between odor compounds and aromatic amino acid metabolism (indole, *p*-cresol, and skatole). However, no significant correlation was found between the total VFA concentration and other odor compounds.

### Animal study

#### Effect of diet on digestibility

After 4 weeks feeding, the body weight of control, CP17, and CP15 were 52.3 kg, 60.3 kg, and 53.1 kg, respectively. The body weight gain of three treatments during four weeks were 20.3 kg, 27.3 kg, and 21.2 kg, respectively. Owing to six pigs in each treatment being fed together with one feeder, the body weight and average daily gain during the feeding experiment were not statistically analyzed.

The ATTD of DM indicated that the CP17 diet resulted in a better ATTD of DM each week (Table 5). Both SID-EAA diets showed higher ATTD of DM than control diet (a high-CP diet without SID-EAA adjustment) after 3 weeks and 4 weeks of feeding (p<0.05). The CP17 diet also resulted in better ATTD of nitrogen during experiment period (quadratic effect of CP level, p<0.01). The ATTD of nitrogen in the control group was lower than that in the other treatment groups after 3 weeks and 4 weeks of feeding (p<0.01), but the quadratic effect of CP level (p<0.01) was shown during feeding period.

#### Effect of diet on odorous compounds in feces

To evaluate the effect of SID-EAA adjustment on odor emission, the concentration of fecal odorous compounds was assayed at week 2 (sampling time was d 11 to d 14) and week 4 (sampling time was d 25 to d 28) (Table 6). At week 2, the highest concentrations of total VFA, NH_3_-N, and indole were observed in the high CP diet without adjustment (control diet). The CP17 diet resulted in similar *p*-cresol and skatole concentrations to the control diet at week 2. However, after feeding for 4 weeks, the concentration of all odorous compounds from amino acid metabolism was lower in pigs fed diets with SID-EAA adjustment (CP17 and CP15). It should be noticed that diet CP level showed linear (p<0.001) and quadratic (p = 0.006) effects on skatole concentrate at week 2 and week 4, the skatole emission decreased over 50% when the CP15 diet was fed. This indicated that the odor emission decreased when the diet CP level decreased, especially in the lower CP diet with SID-EAA adjustment.

According to the correlation observed between fermentation products and enzyme activity from *in vitro* experiments, urease and protease activity seem to be closely associated with odor emission in feces. The activities of both enzymes in the fecal samples are also listed in Table 6. The highest protease and urease activities were observed in the group fed the control diet at week 2 and week 4.

### The relationship between enzyme activity and odor emission from nitrogen metabolites

Since the protease and urease activities seem to be closely connected to the odorous compounds related to nitrogen metabolism in both *in vitro* and *in vivo* experiments, the correlation coefficient between urease activity and NH_3_-N was 0.92 and 0.84 in the *in vitro* and *in vivo* experiments, respectively (Table 4). The correlation coefficients between protease activity and measured nitrogen metabolite concentration in the *in vitro* experiment ranged from 0.62 to 0.82. Compared with protease activity, urease activity was more closely related to nitrogen odorous metabolite concentrations in the *in vivo* experiment. In the *in vivo* experiment, it is worth noting that both protease and urease activity were related to the skatole concentration in the fecal sample (r = 0.58 and 0.61, respectively).

## DISCUSSION

### *In vitro* digestion method

The major advantage of *in vitro* digestibility experiments is a decrease in the experimental cost and the number of animals required. Many *in vitro* methods have been established to study the *in vitro* digestibility of animal diets and to predict *in vivo* digestibility. The three-step *in vitro* model of the pig’s gastrointestinal tract, combining enzymatic hydrolysis and dialysis with batch fermentation and fecal inoculation, proceeded well in previous studies [5,10]. However, numerous studies of fermentation evaluation after gastro-intestinal enzyme digestion have applied the flask shaking method in the gastro-intestinal phase [3]. When the whole gastro-intestinal phase *in vitro* digestion proceeds in sealed containers, the products cannot be removed or absorbed, resulting in a difference from true *in vivo* gastrointestinal tract digestion and absorption in the animal. While many studies have applied filtration or acid precipitation to remove the soluble part of digesta after flask digestion, the feedback inhibition of enzymes during the digestion process is still inconclusive [4].

Jaworski et al [14] showed that replacing buffer during the *in vitro* gastro-intestinal digestion process increased the digestion product removal efficiency and led to higher nitrogen digestibility, which could explain the higher *in vitro* DM and CP digestibility observed in the dialysis method than in the flask method. The feedback inhibition effect of digestion product to digestive enzymes could be decreased through the dialysis process. Compared to the flask method, the substrates from the dialysis method had a shorter C value (time for 1/2 max gas production) and showed differences among CP level treatments. It implied that the digested residual from the dialysis method had less fermentable materials. Both T_max_ and R_max_ values changed according to the diet CP level after dialysis digestion, and the analysis result also showed the digestion method effect was significant in fermentation rate. This suggested that the removed digestion products affected the fermentation performance, and the dialysis method showed better distinguishability to different levels of protein diets. Bauer et al [11] suggested that the degree of feedstuff digestion affects the C, T_max_, and R_max_ during fermentation, and while flask digestion may show good results for single feedstuff evaluation, it showed a defect in evaluating the fermentation potential of a mixed diet.

According to the fermentation results, the individual VFA and the total VFA concentrations among the different CP level diets were very similar after flask digestion. This implies that fermentable carbohydrates in these digestion residues showed no difference. The soluble carbohydrate and fiber content in the digestion residue affects the Ac/Pr ratio [14], and the level of VFA production during fermentation can provide a good prediction of the degree of fermentation of dietary carbohydrates. However, residuals from the flask digestion method showed a low ability to distinguish between the fermentation products, especially in the predictive ability of VFA composition. A previous study comparing *in vitro* and *in vivo* digestion results indicated that the flask digestion method was not correlated with *in vivo* digestibility data obtained from fecal samples [15]. In this study, the *in vivo* DM digestibility coefficients of the CP17 and CP 15 diets ranged from 70.6% to 78.9%, and the *in vivo* nitrogen digestibility ranged from 87.3% to 93.0%. In terms of *in vitro* digestibility results, the results from the dialysis method showed closer relation to the *in vivo* data, but the lower CP digestibility after flask digestion led to more nitrogen compounds being available for subsequent fermentation, and higher undegraded diet proteins flowing into the colon resulted in the higher odor emission in feces [1]. In this study, the decrease in the GI phase CP digestibility also resulted in higher ammonia production after fermentation. This suggests that the flask method may not provide exact results among diets with similar feedstuff composition.

The lower fecal esterase and amylase activities in the *in vitro* study may be due to the high digestibility of lipids and starch during the *in vitro* GI phase digestion. According to the data on enzyme activity change during fermentation and the diet composition, esterase activity seems to have no correlation with the dietary fat levels. The diversity in amylase activity is likely related to the remaining starch (composition of corn in diet) after 12 h fermentation, but showed no difference after 24 h. Based on the results of *in vitro* fermentation product correlation assay, protease and urease activities could be predictors for NH_3_-N emission. However, urease seems to be more sensitive to the final NH_3_-N concentration in this study. A previous study on ureolytic activity and NH_3_-N concentration in pig feces also indicated that urea hydrolysis rates provide a basis for more accurate prediction of ammonia volatilization rates from animal production [16]. It suggested that the esterase and amylase activities could be a parameter for residual lipid and starch digestion during fermentation, respectively. Additionally, Protease and urease could be parameters for residual nitrogen metabolism during fermentation. Macfarlane and Gibson [17] also indicated that at least 12 families of common bacteria in the intestine could produce urease. An intestinal microbiota study showed that Lactobacillales, Lachnospiraceae, and Streptococcaceae groups were positively associated with fecal protease activity across the entire study population, which was also related to the odorous compounds from protein metabolism products of intestinal microbiota.

Comparing the results of the flask and dialysis methods, the urease activity results measured using the dialysis method were more closely related to the NH_3_-N concentration than the flask method. The effect analyzed by orthogonal polynomial contrast also showed the change of urease greatly resembled NH_3_-N emissions. This implies that urease activity may have increased reliability as a predictive parameter for NH_3_-N emissions when dialysis digestion is applied before fermentation.

Analyzing the VFA, NH_3_-N, enzyme activity, and digestibility data between the two *in vitro* digestion methods, it can be suggested that the fermentation residual substrates from the dialysis method were more similar to those in *in vivo* digestion. The VFA and NH_3_-N results indicated that both the CP level and digestion method affected the fermentation products. However, the dialysis method showed better distinguishability with respect to diet CP-level adjustment. In terms of three-step digestion–fermentation *in vitro* evaluation, the flask method may lead to vague end-point product characteristics after fermentation by fecal inoculation.

### *In vivo* experiment

Li et al [18] indicated that decreasing dietary CP levels with EAA supplementation could maintain the growth performance of growing pigs. Furthermore, some studies also reported that a lower CP (decreased to 2% to 4%) diet with SID-EAA adjustment improved the growth performance of growing pigs [2,19]. Le Bellego et al [19] indicated that a 4% decrease in dietary CP level reduced N-excretion by 37% but did not affect growth and carcass composition as long as the ratios between essential amino acids and net energy are kept optimal.

The VFA performance of the fecal samples indicated that the control group had the highest total VFA concentration during the experimental period, which suggested that the available fermentation substrate was abundant in the control group. Lower DM and nitrogen digestibility in the control group may be a reason for this result. Butyrate was identified as a major energy source for the intestines, and up to 95% of microbial-produced butyrate has been estimated to be consumed by the colon [14].

Many feeding studies have reported a reduction in NH_3_ emissions by decreasing the CP level of the diet. Kay and Lee [20] reported up to approximately 47% reduction in NH_3_ emissions in pigs fed with low dietary CP (reduced from 21% to 15%) during the growing phase. Otto et al [21] also reported a 49% decrease in NH_3_ emissions when growing pig dietary CP was reduced from 15% to 12%. In this study, decreasing the CP level with SID-EAA adjustment resulted in a better effect on NH_3_-N reduction. Compared to the control diet, the CP17 and CP15 diets resulted in a 48% and 53% reduction in NH_3_-N, respectively, after 2 weeks of feeding. When urine meets fecal matter, urinary urea is converted back to NH_3_-N via the action of bacterial urease present in the feces. Lower urease activity was observed in the fecal samples from the CP17 and CP15 feeding groups, suggesting that when urine was mixed with these feces, NH_3_-N production could be lowered. The NH_3_-N reduction with lower urease and protease activities resulting from low CP diet was in accordance with the *in vitro* results of this study. The fecal metabolite correlation result also indicated that protease and urease activities were closely related to NH_3_-N production. The NH_3_-N reduction data in the present study suggest that urease and protease showed good predictability with fecal NH_3_-N emissions.

Among fecal odor compounds, phenols, indoles, and branched chain fatty acids (BCFA) showed greater odor intensity and annoyance with a lower odor detection threshold than others [1]. Indole, *p*-cresol, and skatole account for over 90% of the odor activity among all volatile organic compounds resulting from the pig production process [16]. In particular, *p*-cresol is mostly responsible for the overall odor impact from volatile organic compounds emissions [20]. Compared to the control diet, the CP15 diet decreased p-cresol production by over 65% in fecal samples, but the CP17 diet showed less effect on reducing *p*-cresol. A previous, long-term study [22] on feeding pig with decreasing CP also indicated that a low-CP diet (CP levels reduced by 4% of normal) decreased the concentrations of phenol and skatole, but it had no effect on decreasing the concentration of p-cresol even after 77 days of feeding.

Cho et al [23] indicated that a decrease of 2.5% CP in the diet (from 20% to 17.5%) had no effect on the reduction of skatole and *p*-cresol in slurry, but it decreased the indole emission. However, when the CP content decreased to 15%, all odor compounds were reduced after four weeks of feeding. The odor compound performance in the study of Cho et al [23] was similar to the present *in vivo* study and it might be associated with changes in bacterial communities, especially in the roles in protein metabolism. According to a previous bacterial community and abundance assay, Bacteroides and Clostridium were more abundant in the lower CP group than in the higher CP group. These bacteria produce lesser amounts of short chain fatty acid, BCFA, and indole during undigested protein degradation [24]. Wong et al [25] also indicated that the Clostridiaceae family is closely related to tryptophanase and *p*-cresol production enzymes. The correlation result in the present study indicated that protease and urease activities showed moderate to good correlation with skatole, but no strong relationship was observed between these enzyme activities and the other two protein metabolic compounds (*p*-cresol and indole).

Compared with the control diet, the CP17 diet in the present study still showed a reduction in protein-related odor compounds. This may be explained by the balanced amino acid composition in the feed which, in turn, could have resulted in a reduced flow of undigested protein to the colon for fermentation. Recharla et al [26] indicated that the administration of a pig diet formulated with the ideal protein content improves gut fermentation and reduces odor by modulating the gut microbial community. The odor compound reductions in the present study are in line with the previous studies on lower CP-diet; the lower CP-diet with SID-EAA adjustment also contributed to odor emission. Hobbs et al [27] indicated that compared to growing pigs fed on diet with 21% CP, the 14% CP diet plus EAA resulted in an approximately 40% decrease in nitrogen excretion and also reduced the concentrations of most odorants in the slurry. An *in vitro* study by Turner et al [28] also showed that NH_3_ emissions from manure reduced by over 75% by decreasing the dietary CP level in the growing phase diet from 16% to 12% and adding EAA.

### Predictability of *in vitro* markers for *in vivo* experiments

To evaluate the effect of feed on animals more efficiently, establishing a suitable *in vitro* evaluation model could be a good alternative. However, the effective markers or parameters to predict *in vivo* results using the *in vitro* assay procedure should be selected first.

Based on the correlation between the results of *in vitro* and *in vivo* odor compound emissions, a further assay of the ability of protease and urease as odor emission markers was performed in the present study. All assay results of the correlation between fecal enzyme (protease or urease) and odor compound emissions implied that both protease and urease showed a positive correlation with all tested compounds, especially in NH_3_-N and skatole emission. However, the *in vivo p*-cresol and indole emissions showed less sensitivity to increasing protease or urease activity. According to the correlation coefficients between fecal parameters in this study, protease and urease activities are closely related to the odor emission from nitrogen metabolism compounds. Both protease and urease activities seemed to be valuable markers for predicting *p*-cresol concentration in the *in vitro* experiment, but not consistent with the *in vivo* experiment.

Although the products from the fermentation of carbohydrate and protein digestion residues provided the direct odor compound formation level *in vitro*, the absorption of fermentation products, especially VFA, could not be simulated by *in vitro* tests. Zhang et al [29] indicated that high-protein diets increased the microbial fermentation of nitrogen compounds and increased the concentrations of metabolites derived from microbial AA metabolism, especially in the distal part of the intestine. The concentration of end-products in feces may not directly reflect microbial catabolic activity as the concentration depends on the rates of production and absorption. A study on microbial composition and catabolic activity in the colon indicated that both Clostridiaceae and Enterobactetiaceae are matched with bacterial families, containing urease, tryptophanase, and *p*-cresol production enzymes [25]. This suggests that microbial enzyme activity might be a potential marker for odor product formation.

A previous *in vivo* study reported that microbial urease could be applied as a marker for the gut microbiome, as well as for evaluating fecal nitrogen metabolites [25]. According to the correlation analysis results between enzyme activity and *in vitro*/*in vivo* odor products, both protease and urease allowed good evaluation of odor compound formation when the diet CP levels changed in the present study. The *in vitro* enzyme activity assay trends also greatly resembled the *in vivo* results, which implied that the *in vitro* enzyme assay might be connected to the *in vivo* results. Some previous studies have described that *in vitro* studies can be useful for investigating microbial capabilities in this respect [6,26]. The application of pig feces as an inoculum for *in vitro* gas production evaluation has been conducted to investigate the fermentation of different protein sources, and batch culture studies with human fecal microbiota have also provided an effective metabolite profile of peptides and AA fermentation [30].

## CONCLUSION

In conclusion, a suitable GI phase *in vitro* digestion by dialysis followed by fecal inoculation fermentation was established in the present study. To evaluate the emission impact of pig diets, the accuracy and convenience of evaluation could be improved by applying the present *in vitro* experimental models to investigate odor and nitrogen waste emissions before animal experiments. The diet selected after *in vitro* evaluation could be fed to animals, and the growth performance could be estimated for future proof. This could result in reduced time and costs in animal experiments. The *in vitro* and *in vivo* enzyme activity assays could provide a simple and effective estimation tool for odor emission prediction while adjusting the diet formula.

## Figures and Tables

**Table 1:** Diet composition of experiment diets for growing pig (dry matter basis)^1)^

Items	Control^2)^	CP17^2)^	CP15^2)^	CP13^2)^
Ingredients (%)				
Corn, yellow dent	67.60	65.60	67.40	75.20
Corn distiller's dried grains with solubles	4.20	5.00	6.00	0.20
Soybeans, full fat	2.50	3.80	4.40	2.80
Soybean meal, CP 48%	17.30	12.30	5.10	3.10
Wheat bran	2.00	6.05	9.30	10.91
Soybean oil	0.70	1.00	1.06	0.55
Fish meal	2.00	2.00	2.00	2.00
L-Lys·HCl (78.8%)	0.09	0.40	0.62	0.75
DL-Met	0.04	0.04	0.09	0.15
L-Thr	-	0.11	0.19	0.27
L-Trp	-	0.04	0.08	0.10
L-Val	-	-	0.09	0.20
L-Ileu	-	-	0.07	0.16
L-Leu	-	-	-	-
Calcium phosphate (monobasic)	1.24	1.43	1.20	1.25
Limestone	0.83	0.73	0.90	0.86
Sodium chloride	0.50	0.50	0.50	0.50
Vitamin and mineral premix^3)^	1.00	1.00	1.00	1.00
Calculated composition				
Net energy (kcal/kg)	2,474	2,473	2,485	2,489
Crude protein (%)	18.00	17.00	15.00	13.00
Analyzed composition (%)				
Dry matter	87.40	87.20	86.90	87.00
Crude protein	17.90	17.10	15.02	13.20
Crude fat	4.28	3.39	3.90	4.08
Neutral detergent fiber	11.92	13.27	14.28	12.81
Acid detergent fiber	5.41	5.78	6.01	4.92
Ash	3.43	3.52	3.50	3.42

CP, crude protein.

1) Diets used in the *in vivo* feeding experiment and i*n vitro* vs *in vivo* comparing test: Control, CP17, and CP15; Diets used in the *in vitro* digestion experiment: CP17, CP15, and CP13.

2) Control = 180 g CP/kg without essential amino acid (EAA) adjusted; CP17 = 170 g CP/kg; CP15 = 150 g CP/kg; and CP13 = 130 g CP/kg; all three diets were standardized ileal digestible (SID)-EAA adjusted.

3) Premix provided per kilogram of diet as following: vitamin A, 6,000 IU; vitamin D_3_, 800 IU; vitamin B_12_, 0.02 mg; vitamin E, 20 IU; vitamin K_3_, 4 mg; vitamin B_1_, 4 mg; pantothenic acid, 16 mg; niacin, 30 mg; pyridoxine, 1 mg; folic acid, 0.5 mg; biotin, 0.1 mg; Fe (FeSO_4_·7H_2_O), 140 mg; Cu (CuSO_4_·5H_2_O), 7 mg; Mn (MnSO_4_), 20 mg; Zn (ZnO), 70 mg; I (KI), 0.45 mg.

**Table 2:** The effect of diet protein adjustment and digestion methods on fermentation parameters after 48 h fermentation^1)^

Inoculation	Flask			Dialysis			SEM	p-value^2)^				
		
	CP17	CP15	CP13	CP17	CP15	CP13		M	CPL	CPQ	MCPL	MCPQ
Gas production parameters^3)^												
A (mL)	152.31	144.31	145.04	145.25	139.62	143.96	1.60	0.071	0.066	0.168	0.548	<0.001
B (h^−1^)	1.68	1.57	1.70	1.96	1.99	1.95	0.05	<0.001	0.933	0.971	0.029	<0.001
C (h)	10.54	11.91	10.56	6.54	7.44	9.43	0.51	<0.001	0.005	0.501	0.001	<0.001
T_max_ (h)	4.67	4.46	4.38	3.65	4.46	5.09	0.15	0.501	0.026	0.997	0.001	<0.001
R_max_ (h^−1^)	8.88	7.49	8.89	14.26	12.37	9.71	0.62	<0.001	<0.001	0.429	<0.001	<0.001
*In vitro* disappearance %^4)^												
GI phase DM	66.35	61.12	59.21	70.57	59.85	57.53	0.34	<0.001	<0.001	0.004	0.006	0.009
Total DM	73.41	67.63	65.18	77.15	74.69	71.09	0.19	<0.001	<0.001	0.169	<0.001	0.224
GI phase CP	60.76	65.94	67.53	65.37	71.58	75.24	0.18	<0.001	<0.001	0.004	<0.001	0.003
Total CP	88.67	87.12	87.64	91.44	90.59	91.62	0.06	<0.001	<0.001	<0.001	<0.001	0.008

SEM, standard error of the means; GI, gastric-intestinal; DM, dry matter; CP, crude protein.

1) CP17 = 170 g CP/kg; CP15 = 150 g CP/kg; and CP13 = 130 g CP/kg; all three diets were standardized ileal digestible essential amino acid (SID-EAA) adjusted.

2) M, digestion methods; CPL, linear effect of CP level; CPQ, quadratic effect of CP level; MCPL, linear interaction between CP level and digestion method; MCPQ, quadratic interaction between CP level and digestion method.

3) A, asymptotic gas production; B, switching characteristic of the curve; C, the time at half of the asymptote has been reached; T_max_, the time at R_max_ occurs; R_max_, the maximum rate of gas production.

4) GI phase, enzyme digestion with pepsin (in G-buffer) and pancreatin (in I-buffer); Total, GI phase+fecal inoculation fermentation for 48 h.

**Table 3:** The effect of diet protein adjustment and digestion methods on fermentation product and enzyme activity during 48 h fermentation^1)^

Inoculation	Flask			Dialysis			SEM	p-value^2)^				
		
	CP17	CP15	CP13	CP17	CP15	CP13		M	CPL	CPQ	MCPL	MCPQ
	----------------------------------------------------------------------------------- 12 h -----------------------------------------------------------------------------------------											
pH	6.29	6.48	6.06	6.1	6.03	6.01	0.03	<0.001	<0.001	<0.001	<0.001	0.518
VFA												
Acetic acid (mM)	6.61	6.6	6.63	7.76	10.05	10.95	0.11	<0.001	<0.001	0.004	<0.001	<0.001
Propionic acid (mM)	6.40	6.32	6.36	4.99	4.55	3.44	0.15	<0.001	<0.001	0.553	<0.001	<0.001
Butyric acid (mM)	3.35	3.19	3.27	4.96	4.53	4.40	0.09	<0.001	0.005	0.271	0.064	0.124
Total VFA (mM)	16.36	16.11	16.26	17.7	19.14	18.79	0.24	<0.001	0.063	0.295	0.010	0.143
NH_3_-N (mg/dL)	85.8	116.76	108.5	61.55	49.8	31.31	5.85	<0.001	0.069	0.082	<0.001	<0.001
Enzyme activity^3)^												
Esterase	3.68	5.33	3.35	3.79	3.44	2.35	0.12	<0.001	<0.001	<0.001	<0.001	<0.001
Amylase	2.02	1.97	2.84	2.53	1.58	3.52	0.3	0.328	0.002	0.013	0.230	0.681
Urease	3.62	5.15	4.74	2.01	2.69	1.24	0.22	<0.001	<0.001	<0.001	0.001	<0.001
Protease	2.71	2.89	2.91	2.59	2.74	2.45	0.10	0.013	0.283	0.292	0.238	0.208
	---------------------------------------------------------------------------------- 24 h ----------------------------------------------------------------------------------											
pH	5.62	5.61	5.51	4.99	5.14	4.98	0.03	<0.001	0.002	0.005	0.050	0.637
VFA												
Acetic acid (mM)	12.7	12.66	12.74	16.45	18.09	19.22	0.25	<0.001	<0.001	0.878	<0.001	<0.001
Propionic acid (mM)	12.29	12.14	12.2	9.13	7.72	6.18	0.26	<0.001	<0.001	0.997	<0.001	<0.001
Butyric acid (mM)	6.03	5.75	5.88	8.18	7.40	6.80	0.14	<0.001	<0.001	0.543	0.004	0.007
Total VFA (mM)	31.02	30.55	30.82	33.76	33.22	32.21	0.44	<0.001	0.176	0.986	0.262	0.368
NH_3_-N (mg/dL)	90.73	158.39	105.3	93.85	77.24	51.51	3.65	<0.001	<0.001	<0.001	<0.001	<0.001
Enzyme activity^3)^												
Esterase	3.88	4.17	3.69	2.89	3.13	2.00	0.16	<0.001	<0.001	0.008	0.098	0.047
Amylase	4.97	5.04	5.01	5.04	5.02	5.02	0.01	0.054	0.268	0.334	0.005	0.084
Urease	3.61	7.19	5.06	3.36	2.87	2.37	0.20	<0.001	<0.001	<0.001	<0.001	<0.001
Protease	2.68	2.74	2.68	2.71	2.83	2.52	0.10	0.843	0.216	0.317	0.475	0.519
	---------------------------------------------------------------------------------- 48 h ----------------------------------------------------------------------------------											
pH	5.74	5.93	5.81	5.54	5.45	5.22	0.07	<0.001	0.064	0.214	0.035	0.023
VFA												
Acetic acid (mM)	15.42	14.55	14.92	20.90	23.27	24.85	0.88	<0.001	<0.001	0.914	<0.001	<0.001
Propionic acid (mM)	14.14	14.59	13.72	11.94	9.99	8.00	0.51	<0.001	<0.001	0.355	<0.001	<0.001
Butyric acid (mM)	7.80	7.43	7.60	10.57	9.57	8.80	0.25	<0.001	<0.001	0.543	0.004	0.007
Total VFA (mM)	37.36	36.57	36.24	43.41	42.83	41.64	0.65	<0.001	0.019	0.995	0.618	0.767
NH_3_-N (mg/dL)	465.71	398.87	403.00	247.8	136.9	102.9	35.24	<0.001	<0.001	0.523	0.282	0.566
Enzyme activity^3)^												
Esterase	4.90	6.05	5.58	4.62	4.09	3.39	0.44	0.560	0.001	0.614	0.106	0.105
Amylase	1.69	2.55	2.76	2.53	1.84	1.83	0.32	0.331	0.864	0.998	0.032	0.055
Urease	9.79	6.97	7.11	7.81	4.03	3.26	0.48	<0.001	<0.001	0.021	<0.001	0.001
Protease	3.15	3.13	3.14	4.19	3.15	3.11	0.09	0.001	0.001	0.445	<0.001	0.002

CP, crude protein; VFA, volatile fatty acid; SEM, standard error of the means.

1) CP17 = 170 g CP/kg; CP15 = 150 g CP/kg; and CP13 = 130 g CP/kg; all three diets were standardized ileal digestible essential amino acid (SID-EAA) adjusted.

2) M, digestion methods; CPL, linear effect of CP level; CPQ, quadratic effect of CP level; MCPL, linear interaction between CP level and digestion method; MCPQ, quadratic interaction between CP level and digestion method.

3) Unit of enzyme activity: Esterase, *p*-Naphthol μmole/mL/min; Amylase, 100×glucose nmole/mL/min; Urease, μmole NH_3_/mL/min; Protease, μg azo-group/mL/min.

**Table 4:** Correlation coefficients (r) between fermentation products^1)^ of *in vitro* fermentation samples (upper half) and *in vivo* fecal samples (lower half)

r	Ac^1)^	Pr^1)^	Bu^1)^	TVFA^1)^	NH_3_-N	*p*-Cresol	Indole	Skatole	Protease	Urease
*In vitro* fermentation samples (substrates from dialysis digestion method)										
Ac	-	0.365^*^	0.316	0.820^***^	0.526^**^	0.074	0.606^***^	0.191	0.424^**^	0.466^**^
Pr	0.300	-	−0.550^*^	0.864^***^	−0.020	−0.338	−0.555^**^	0.097	0.023	−0.022
Bu	−0.006	0.847^***^	-	0.801^***^	0.004	0.362^*^	0.770^***^	0.262	0.693^***^	0.035
TVFA	0.628^**^	0.916^***^	0.745^***^	-	0.255	0.257	0.731^***^	0.213	0.221	0.232
NH_3_-N	0.329	−0.236	−0.322	−0.063	-	0.800^***^	0.591^***^	0.749^***^	0.805^***^	0.918^***^
*p*-Cresol	−0.162	0.220	0.311	0.133	0.424	-	0.709^***^	0.732^***^	0.617^**^	0.876^***^
Indole	0.372	0.581^*^	0.488^*^	0.627^**^	0.468	0.702^***^	-	0.819^***^	0.820^***^	0.523^**^
Skatole	−0.082	0.144	0.299	0.131	0.601^**^	0.771^***^	0.661^**^	-	0.629^***^	0.668^***^
Protease	−0.022	−0.602^**^	−0.497^*^	−0.470^*^	0.859^***^	0.315	0.105	0.583^*^	-	0.905^***^
Urease	0.007	−0.513^*^	−0.464	−0.402	0.843^***^	0.275	0.110	0.613^**^	0.967^***^	-
*In vivo* fermentation samples										

1) Ac, acetate; Pr, propionate; Bu, butyrate; TVFA, total volatile fatty acids.

* p<0.05;

** , p<0.01;

*** , p<0.001;

without labeled, non-significant.

**Table 5:** Nutrient apparent total tract digestibility of *in vivo* feeding experiment

Item^2)^	Treatment^1)^			SEM	p-value^3)^	
	
	Control	CP17	CP15		L	Q
Apparent total tract digestibility of dry matter (%)						
Week 1 (d 4 to 7)	71.76	73.09	72.67	0.36	0.273	0.216
Week 2 (d 11 to 14)	71.98	78.97	73.09	0.46	0.308	<0.001
Week 3 (d 18 to 21)	70.20	75.99	75.92	0.36	<0.001	0.002
Week 4 (d 25 to 28)	68.12	72.15	70.56	0.51	0.041	0.012
Apparent total tract digestibility of nitrogen (%)						
Week 1 (d 4 to 7)	89.27	89.66	87.43	0.21	0.025	0.007
Week 2 (d 11 to 14)	90.14	93.04	90.46	0.10	0.146	<0.001
Week 3 (d 18 to 21)	88.26	92.75	89.74	0.19	0.004	<0.001
Week 4 (d 25 to 28)	85.05	89.06	87.36	0.17	<0.001	<0.001

CP, crude protein; SEM, standard error of the means.

1) Control = 180 g CP/kg without essential amino acid (EAA) adjusted; CP17 = 170 g CP/kg with standardized ileal digestible essential amino acid (SID-EAA) adjusted; CP15 = 150 g CP/kg with SID-EAA adjusted.

2) Sampling time of feces at the week showed in the brackets.

3) L, linear effect of CP level; Q, quadratic effect of CP level.

**Table 6:** The effect of different crude protein (CP) level on the concentration of volatile fatty acid (VFA) nitrogen related odorous compounds daily emission, and fecal enzyme activity in growing pig feces

Item^1)^	Treatment^2)^			SEM	p-value^3)^	
	
	Control	CP17	CP15		L	Q
	------------------------------------------------------------ Week 2 (d 11 to d 14) ----------------------------------------------------------------					
VFA in fresh faeces (mM)						
Acetic acid	54.73	36.17	44.68	1.71	0.038	0.005
Propionic acid	45.48	37.90	37.99	1.04	0.037	0.206
Butyric acid	29.50	33.22	25.84	0.63	0.186	0.028
Total VFA	129.71	107.29	108.51	2.49	0.006	0.045
Nitrogen related odorous compounds excretion (mg/d/head)						
NH_3_-N	275.84	108.01	122.83	8.66	<0.001	<0.001
Indole	10.43	7.43	5.80	0.22	<0.001	0.199
*p*-Cresol	125.24	119.67	72.64	4.20	<0.001	0.003
Skatole	30.78	29.3	11.65	0.84	<0.001	0.002
Enzyme activity^4)^						
Urease	5.87	5.44	5.42	0.07	0.032	0.241
Protease	5.89	5.55	5.23	0.02	<0.001	0.954
	----------------------------------------------------------- Week 4 (d 25 to d 28) --------------------------------------------------------------------					
VFA in fresh faeces (mM)						
Acetic acid	43.23	41.31	49.05	2.47	0.523	0.534
Propionic acid	32.71	18.24	28.61	0.98	0.203	0.001
Butyric acid	26.65	11.16	21.83	0.75	0.104	<0.001
Total VFA	102.59	70.72	99.49	3.16	0.823	0.001
Nitrogen related odorous compounds excretion (mg/d/head)						
NH_3_-N	350.26	263.71	243.14	13.28	0.001	0.902
Indole	8.51	6.15	5.69	0.21	0.001	0.089
*p*-Cresol	122.22	102.18	72.74	3.99	0.001	0.762
Skatole	51.51	21.63	15.23	1.37	<0.001	0.006
Enzyme activity^4)^						
Urease	7.16	6.61	6.17	0.07	<0.001	0.891
Protease	7.13	6.77	6.29	0.02	<0.001	0.317

SEM, standard error of the means.

1) Sampling time of feces at the week showed in the brackets.

2) Control = 180 g CP/kg without essential amino acid (EAA) adjusted; CP17 = 170 g CP/kg with standardized ileal digestible (SID)-EAA adjusted; CP15 = 150 g CP/kg with SID-EAA adjusted.

3) L, linear effect of CP level; Q, quadratic effect of CP level.

4) Urease activity: μmol NH_3_ released/mL/min; Protease activity: mg azo-group released/mL/min.
